# Monoclonal Gammopathy of Renal Significance With Preexisting Connective Tissue Disease: A Case Report

**DOI:** 10.7759/cureus.66046

**Published:** 2024-08-02

**Authors:** Huang Hin Chin, Ler Yi Lee, Thurgaashini Kethiravan, Hemlata Kumari Gnanasegaram, Nur Afrina Muhamad Hendri, Ban Chai Peng

**Affiliations:** 1 Nephrology, Hospital Melaka, Melaka, MYS; 2 Pathology, Hospital Kuala Kumpur, Kuala Lumpur, MYS; 3 Electron Microscopy Unit, Institute of Medical Research, Kuala Lumpur, MYS

**Keywords:** case report, hematology, nephrology, sle, mgrs, monoclonal gammopathy of renal significance

## Abstract

Monoclonal gammopathy of renal significance (MGRS) has lately drawn the interest of physicians and pathologists due to the ability of these monoclonal proteins to cause end-organ damage. The early detection of this monoclonal protein along with hematological studies and renal biopsy are essential to establish the associated nephropathological diagnosis. We herein describe the case of a patient with MGRS and the diagnostic entity involved. She responded well to the treatment as co-managed by a multidisciplinary team of nephrologists, hematologists, and renal pathologists.

## Introduction

Plasma cell dyscrasia is a proliferation of either premalignant or malignant clone of plasma cells which is usually associated with the production of a monoclonal protein. There are several subtypes of plasma cell dyscrasia such as monoclonal gammopathy (MG) of undetermined significance, malignant monoclonal gammopathies, malignant lymphoproliferative disorders, heavy chain disease, and immunoglobulin deposition disease [1]. These immunoglobinopathies which consist of light and heavy chains can be usually detected in the serum or urine samples of a patient. They frequently entail a heterogeneous set of overlapping clinical signs and tedious laboratory investigations, thus making them challenging to diagnose [1]. This is a rare case of a young female patient with underlying connective tissue disease who has persistent proteinuria despite being on optimal treatment. She underwent further laboratory testing which led to the findings of monoclonal gammopathy of renal significance (MGRS).

## Case presentation

This is a case of a 42-year-old female with underlying systemic lupus erythematosus (SLE) with extrarenal manifestations (arthritis, serositis, mucocutaneous and oral ulcers), rheumatoid arthritis, hypothyroidism, and gastroesophageal reflux disease. Her immunological tests such as anti-nuclear antibodies (ANA) and double-stranded DNA (DsDNA) were positive (1:100 homogeneous). She has been under rheumatology team follow-up since January 2010. Yearly OGDS (oesophagogastroduodenoscopy) showed erosive gastric mucosa with esophagitis while colonoscopy showed proctocolitis and ileitis. She worked as a librarian and married with two children. Her disease activity was relatively stable with no frequent flares.

In July 2022, routine monitoring noted significant proteinuria of 1.82 g/24 h. She denied symptoms of frothy urine, peripheral limb swelling, or anasarca. Her blood pressure remained normotensive. There were no ingestions of supplements or traditional medications. Physical examination was unremarkable. Other blood investigations were normal including serum albumin and thyroid function tests. Her tumor markers screening was negative. A referral to the nephrology team was made and she was counseled for renal biopsy. The renal biopsy was performed in November 2022, which showed minor glomerular changes with negative immunofluorescence. Unfortunately, no electron microscopy examination was performed. She was treated for minimal change disease with a background of SLE (lupus podocytopathy). She was given glucocorticoids and angiotensin receptor inhibitors as part of the treatment.

**Figure 1:**
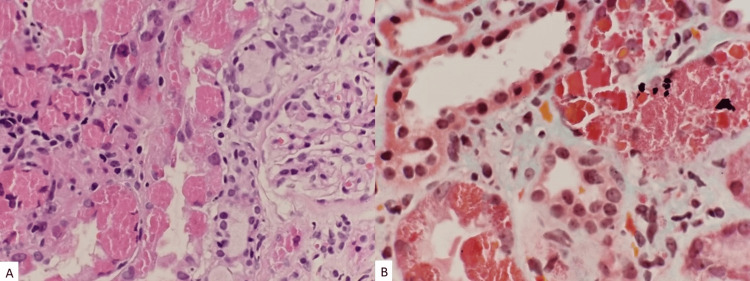
(A, B) Light microscopy and special stain studies. In both (A) and (B), the proximal tubules throughout the core exhibit epithelial cells that are enlarged containing intracytoplasmic material/inclusions which are brightly eosinophilic in H&E stains (A) and fuchsinophilic in Masson trichrome stain (B). These intracytoplasmic materials show variation in sizes with absence of crystal formations. As for the distal tubules, besides seeing intraluminal desloughed cells containing intracytoplasmic material, the lining epithelium of the distal cells do not harbor any significant intracytoplasmic material. (A) H&E stain (original magnification, x400) and (B) Masson trichrome (original magnification, x600).

**Figure 2:**
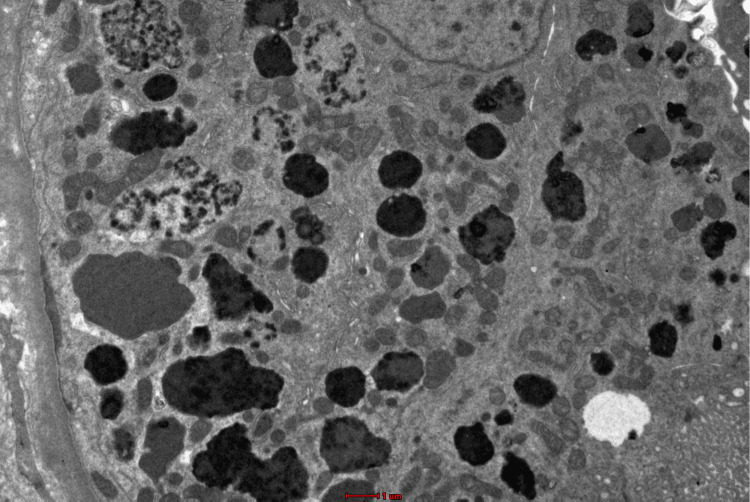
Electron microscopy images demonstrate proximal tubules enlarged with numerous dysmorphic lysosomes showing mottled granules with some exhibiting pearl string-like electron-dense granules within areas of lucency (electron microscopy; original magnification, x2700 nm).

Unfortunately, her symptoms persisted despite being on medications. Renal impairment started in September 2023 with a serum creatinine value of 127 umol/L. Basic routine blood and urine tests were conducted, which showed UFEME (urine full examination, microscopic examination) of protein 4+ and RBC 2+, while serum liver function test showed total protein 58 g/L, albumin 39 g/L, and globulin 19 g/L. Her quantified proteinuria was 2.84 g/24 h. Otherwise, she remained well with no complaints. She was reinvestigated with serum protein electrophoresis, which showed oligoclonal bands with significantly raised serum free Kappa light chain of 1430.00 mg/L (reference range 6.70-22.40 mg/L), while urine protein electrophoresis noted Kappa light-chain paraproteinuria of 4.37 g/L. Thus, a second renal biopsy (Figure 1) was recommended with an electron microscopy study. The renal biopsy showed Kappa positivity at the tubular epithelium with IgG, IgA, C3, C1q, and Lambda negativity at the glomerular capillary wall. Congo red stain did not demonstrate amyloid deposits. Electron microscopy examination (Figure 2) revealed normal mesangial matrix and cellularity with podocytes showing intact foot processes. Membrane-bound vacuoles and aggregated non-membrane-bound bundles of parallel-to-random non-branching fibrils ranging a thickness of 9.42-11.2 nm are seen within the cytoplasm. A diagnosis of non-crystalline light-chain proximal tubulopathy with monoclonal Kappa light chain restriction was made.

She underwent bone marrow aspiration and trephine biopsy as well. However, due to suboptimal bone marrow sampling, it showed 0.1% plasma cells with CD138 positivity. Cytogenetic analysis showed no excess plasma cells or lymphomatous infiltration. After consultation with a hematologist, she agreed to undergo a chemotherapy regime of bortezomib + dexamethasone + thalidomide. Her renal function improved gradually with the latest serum creatinine showing 84 umol/L and 24-h urine protein showing 0.94 g/day, respectively. She is still under routine hematology follow-up with the long-term aim of stem cell transplantation (SCT).

## Discussion

MG is a premalignant plasma cell dyscrasia marked by increased monoclonal immunoglobulin synthesis, which is most likely caused by continuous antigenic stimulation resulting in aberrant B-cell clonal growth. The accumulated B-cell or plasma cell clone within the bone marrow causes an increase in monoclonal immunoglobulin synthesis [2]. The International Kidney and Monoclonal Gammopathy Research Group first introduced the term MGRS in 2012, which is defined as a clonal proliferative disorder of B cells or plasma cells that produces a nephrotoxic monoclonal immunoglobulin and does not meet the hematologic criteria for treatment of a specific malignancy [3]. This monoclonal protein features as light chain, heavy chain, or immunoglobulins. The rationale for establishing this new category stemmed from the fact that there are patients with MG but no organ involvement, as opposed to patients who are at risk of developing kidney disease, as these latter patients have a high level of morbidity/mortality due to the systemic organ dysfunction caused by the monoclonal protein. Thus, early identification and definition of the kidney lesion is imperative to begin treatment and stop the secretion of the paraprotein to prevent the evolution to end-stage renal disease and enhance both renal and patient survival. Based on the seventh report of The Malaysian Registry of Renal Biopsy, the incidence of the presence of light/heavy chains in renal biopsy is only 0.1% [4].

The clinical presentation of MGRS varies as it often involves renal and extrarenal conditions such as neuropathy, dermopathy, and eye disorders. Deregulation of the alternative complement pathway or the release of endothelial growth factors is thought to have caused indirect lesion deposition in the vascular endothelium, resulting in cutaneous lesions, thrombotic microangiopathy, and the POEMS syndrome. Renal manifestation is derived from various degrees of proteinuria, tubulopathies, and subsequently renal failure [3]. In this case, the patient had worsened renal function with heavy proteinuria, which warranted renal biopsy for diagnostic purposes.

The diagnosis of MGRS remained challenging as it involved hematological and renal evaluation. As the diagnosis is frequently delayed, a high level of clinical suspicion, as well as a multidisciplinary team approach involving hematologists, nephrologists, and renal pathologists, is required. Hogan JJ et al. suggested four approaches in diagnosing MGRS, namely (1) renal biopsy for identification of the pattern of renal parenchymal damage and demonstration of monoclonal protein, if present, (2) identification of the corresponding monoclonal protein in the serum and/or urine, (3) demonstration of the underlying clonal population of cells secreting the monoclonal protein, and (4) characterization of extrarenal manifestations of the clonal disorder [5]. In this case, our patient fulfilled the diagnostic criteria with renal biopsy-proven light chain tubulopathy with monoclonal protein deposits, positive of free light chain (FLC) in serum and urine samplings, and the presence of extrarenal end-organ manifestations.

The latest consensus applied treatment targeting directly at the B-cell or plasma cell clones which involves SCT, immunomodulator (thalidomide, lenalidomide, pomalidomide), proteasome inhibitor (bortezomib, carfilzomib, ixazomib), and monoclonal antibodies (daratumumab, elotuzumab) [3]. Early intervention helps to decrease serum FLCs, which in turn allows a high rate of renal recovery [6]. Therefore, the primary goal in treating MGRS is to arrest the course leading to kidney failure by avoiding irreversible renal and systemic complications attributable to tubulointerstitial fibrosis progression in end-stage kidney disease. In our case, the patient is undergoing a combination of bortezomib + dexamethasone + thalidomide regime. She was scheduled for SCT at our central tertiary hematology hospital located in Kuala Lumpur.

## Conclusions

MGRS is a new diagnostic entity involving a spectrum of clinical and laboratory presentations. We need to have a high index of suspicion and a multidisciplinary approach involving a nephrologist, a hematologist, and a renal pathologist.

## References

[1] International Myeloma Working Group (2003). Criteria for the classification of monoclonal gammopathies, multiple myeloma and related disorders: A report of the International Myeloma Working Group. Br J Haematol.

[2] Seifert M, Scholtysik R, Küppers R (2013). Origin and pathogenesis of B cell lymphomas. Methods Mol Biol.

[3] Alonso-Titos J, Martínez-Esteban MD, López V, León M, Martin-Reyes G, Ruiz-Esteban P, Hernández D (2021). Monoclonal gammopathy of renal significance: Early diagnosis is key. Nefrologia (Engl Ed).

[4] (2022). 7th Report of the Malaysian Registry of Renal Biopsy. https://www.msn.org.my/nrr/7th-report-of-the-malaysian-registry-of-renal-biopsy/.

[5] Hogan JJ, Weiss BM (2016). Bridging the divide: An onco-nephrologic approach to the monoclonal gammopathies of renal significance. Clin J Am Soc Nephrol.

[6] Caravaca-Fontán F, Gutiérrez E, Delgado Lillo R, Praga M (2017). Monoclonal gammopathies of renal significance. Nefrologia.

